# Effect of Zinc Phosphate on the Corrosion Behavior of Waterborne Acrylic Coating/Metal Interface

**DOI:** 10.3390/ma10060654

**Published:** 2017-06-14

**Authors:** Hongxia Wan, Dongdong Song, Xiaogang Li, Dawei Zhang, Jin Gao, Cuiwei Du

**Affiliations:** 1 Institute for Advanced Materials and Technology, University of Science and Technology Beijing, Beijing 100083, China; wanhongxia88@163.com (H.W.); dawzhang@126.com (D.Z.); g.jin@163.com (J.G.); dcw@ustb.edu.cn (C.D.); 2 Aerospace Research Institute of Materials and Processing Technology, Beijing 100076, China; 3 Ningbo Institute of Material Technology and Engineering, Chinese Academy of Sciences, Ningbo 315201, China

**Keywords:** waterborne acrylic coating, zinc phosphate, LEIS

## Abstract

Waterborne coating has recently been paid much attention. However, it cannot be used widely due to its performance limitations. Under the specified conditions of the selected resin, selecting the function pigment is key to improving the anticorrosive properties of the coating. Zinc phosphate is an environmentally protective and efficient anticorrosion pigment. In this work, zinc phosphate was used in modifying waterborne acrylic coatings. Moreover, the disbonding resistance of the coating was studied. Results showed that adding zinc phosphate can effectively inhibit the anode process of metal corrosion and enhance the wet adhesion of the coating, and consequently prevent the horizontal diffusion of the corrosive medium into the coating/metal interface and slow down the disbonding of the coating.

## 1. Introduction

Serious environment problems have directed attention toward water-based coatings because of their low volatile organic compounds VOC advantages [1,2,3]. However, waterborne coatings have performance limitations; moreover, they have been extensively used indoors, where the corrosion environment is below the C3 level [4]. Thus, waterborne coatings need to have improved performance for long-term applications [5,6,7,8]. Acrylic coatings have good barrier properties and ageing resistance, which has attracted much attention [9,10]. Disbonding is the first sign of coating/metal interface corrosion [11,12,13,14], which is the most common form of failure that limits coatings, especially for long-term applications of waterborne coatings [15]. Hence, the ability to resist disbonding characterizes the coating service life, which is the main purpose for developing long-lasting water-based coatings suited for harsh environments [16]. Traditional coatings would exhibit significant cathodic disbonding under different environments that would affect the coating service life. Adding an electrochemically active antirust pigment is a common method for improving the antirust capacity of the coating [17,18]. Therefore, the effect of an electrochemically active antirust pigment on the cathodic disbonding process when added into a waterborne acrylic coating is significant.

Much attention has been given to phosphate antirust pigments for their relative environmental protection [19,20,21,22,23]. Phosphate pigments mainly contain aluminum tripolyphosphate [24] and zinc phosphate [25]. The antirust function and mechanism of zinc phosphate have been previously studied. Bethencourt et al. [26] studied the failure process of a waterborne acrylic zinc phosphate coating using electrochemical impedance spectroscopy (EIS). The corrosive failure of composite coatings in 3.5% NaCl solution were divided into three stages: the first stage is the water diffusion stage, wherein the coating impedance decreases; the second stage involves the zinc phosphate coming in contact with water, wherein a passivation rust resistance effect reaches a certain concentration after water absorption; and the third stage is the start of the shielding decline after the zinc phosphate is exhausted. Yawei Shao et al. [27,28] studied the corrosion inhibition of zinc phosphate in an oil-based epoxy coating using EIS and electrochemical noise, and showed that zinc phosphate provides a recovery effect for coating defects. Currently, researchers [29] found that when the corrosive medium permeates the oil-based epoxy coating/metal interface, zinc phosphate partly dissolves and forms metal substrate phosphate and stabilizes the protective film, which contains γ-Fe_2_O_3_, α-FeOOH, γ-FeOOH, and iron phosphate. Moreover, the protective film contains carboxyl and hydroxyl groups that enhance the combination of coating and metal. Some researchers [30] have proposed that zinc phosphate can form stable iron phosphates and block medium diffusion into the channels and slow down corrosion. However, the effects of zinc phosphate active fillers on the waterborne acrylic cathodic disbonding process, especially involving the interface corrosion mechanism of cathodic and anodic processes, have not been comprehensively studied.

In this paper, we studied the coating/metal interface corrosion process of waterborne acrylic coatings with zinc phosphate using EIS and localized EIS (LEIS). Furthermore, we studied the effects of active pigments on coating/metal interface corrosion and disbonding processes in waterborne acrylic coatings.

## 2. Results

### 2.1. The Coating Adhesion Test 

**Figure 1:**
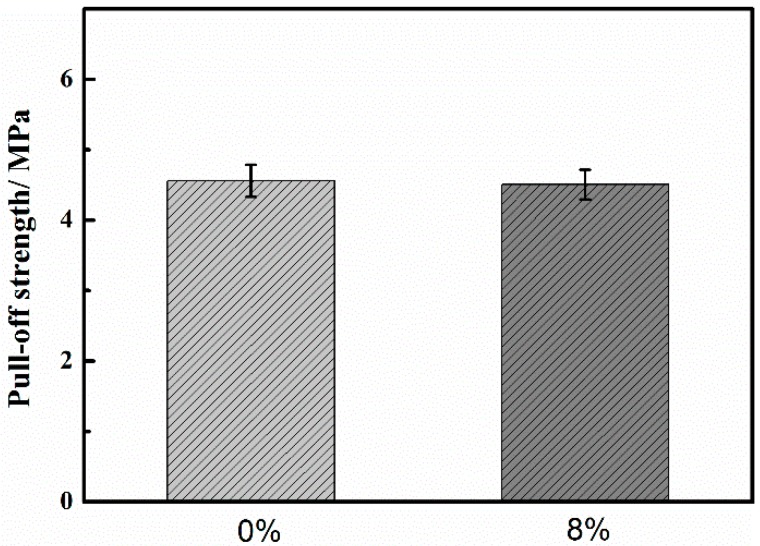
The adhesion values of 0% and 8% zinc phosphate coatings.

**Figure 2:**
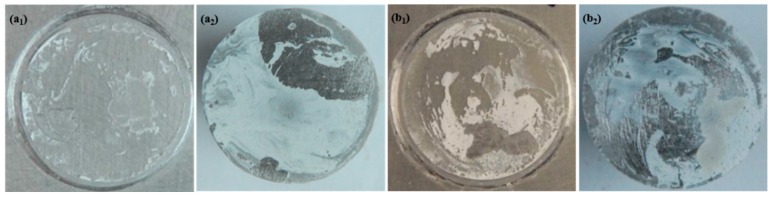
The fractures after the pull-off experiments in (**a_1_**,**a_2_**) 0% and (**b_1_**,**b_2_**) 8% zinc phosphate coatings.

Figure 1 shows the adhesion values of 0% and 8% zinc phosphate coatings. The adhesion values were the same in the 0% and 8% zinc phosphate coatings. Adding zinc phosphate did not affect the initial coating mechanical performance. The coating fractures after the pull-off experiments are shown in Figure 2.

Figure 2 shows the adhesive fracture in the samples without and with 8% zinc phosphate coating. The fracture mode for the 8% zinc phosphate coating after the pull-off experiment, which is the mixture of the coating body and adhesive interlayer fractures, showed strong adhesion. This result is the same with the sample without zinc phosphate coating, only the ratio of the coating body and adhesive interlayer fractures is different.

### 2.2. The Coating Morphology 

**Figure 3:**
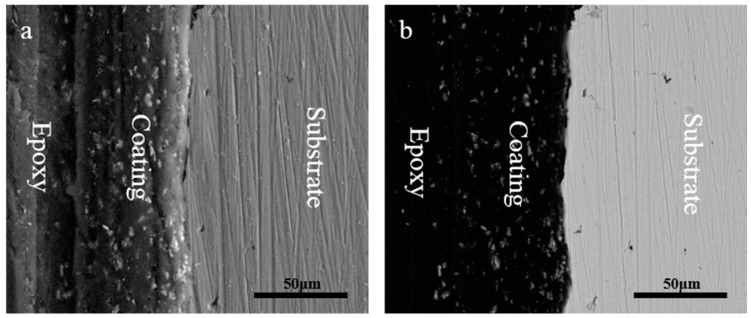
The section morphology of the 8% zinc phosphate coating: (**a**) secondary electron morphology and (**b**) back scattering morphology.

Figure 3 shows the scanning electron microscope (SEM) section of the 8% zinc phosphate coating. Figure 3a shows the secondary electron morphology and Figure 3b shows the scattering electronic morphology. The zinc phosphate coating has a homogeneous dispersion and combined with the resin tightly. Moreover, adding zinc phosphate did not show defects.

**Figure 4:**
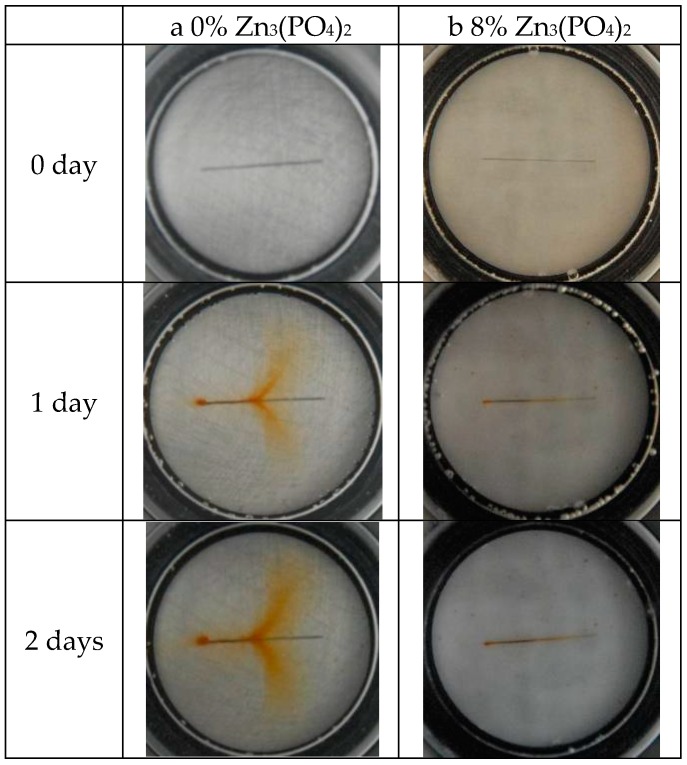

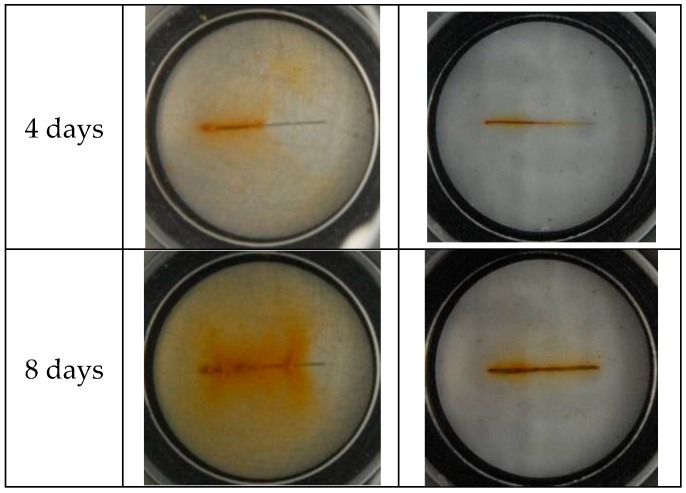
The macromorphology of (**a**) 0% and (**b**) 8% zinc phosphate coating scratches with different immersion days.

Figure 4 shows the macromorphology of the coating without and with 8% zinc phosphate in 3.5% NaCl solution after various immersion days. At the beginning, the 0% and 8% zinc phosphate coating morphologies were the same. However, after two days of immersion, the 0% zinc phosphate coating generated obvious corrosion. The coating generated serious corrosion after four days of immersion, and the defective coating bubbled after eight days. In the 8% zinc phosphate coating, rust occurred after four days of immersion and increased after eight days, but no bubbling was observed.

**Figure 5:**
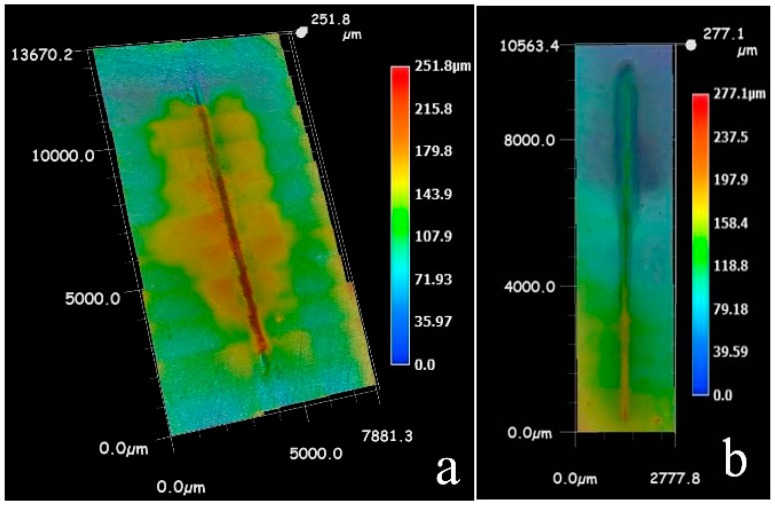
Microstructures of coating with (**a**) 0% zinc phosphate and (**b**) 8% zinc phosphate immersed for eight days in 3.5% NaCl solution.

The microstructures after immersion are shown in Figure 5. The coating defects without zinc phosphate bubbled in 3.5% NaCl solution after eight days, and that with 8% zinc phosphate did not bubble. These results indicated that the coating with zinc phosphate can improve the disbonding resistance.

### 2.3. The Electrochemical Behavior of Zinc Phosphate Coating

#### 2.3.1. Macroscopic Electrochemical Behavior

**Figure 6:**
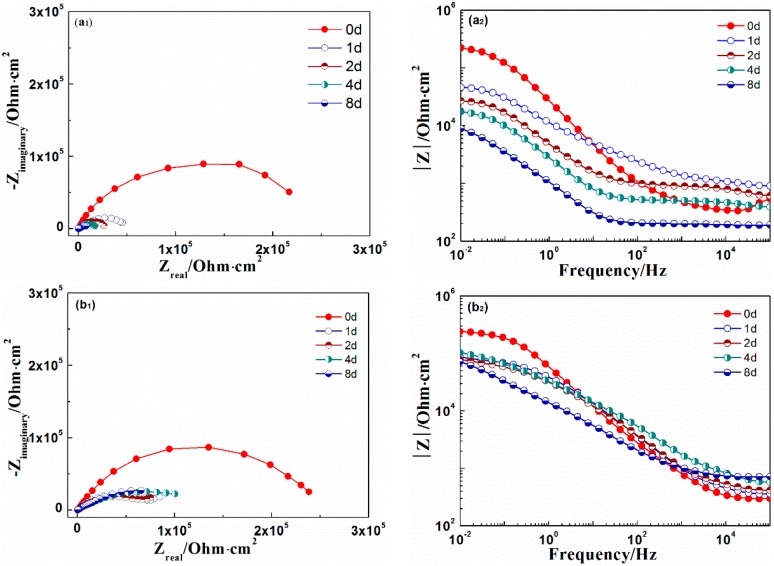
The electrochemical impedance spectroscopy (EIS) of (**a_1_-Nquist**, **a_2_-Bote**) 0% and (**b_1_-Nquist**, **b_2_-Bote**) 8% zinc phosphate coatings.

**Figure 7:**
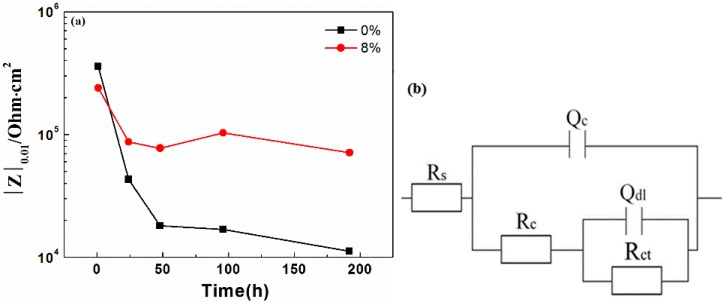
(**a**) Low-frequency impedance value and (**b**) equivalent circuit of EIS of the 0% and 8% zinc phosphate coatings.

Figure 6 shows the EIS results of coatings immersed in 3.5% NaCl solution. The drop in the low frequency impedance value of the 0% zinc phosphate coating indicates the fast corrosion along the coating/metal interface. The high-frequency impedance value of 0% zinc phosphate coating was reduced after two days of immersion, indicating that the corrosive medium in the coating/metal interface was diffused and that disbonding of the coating occurred. However, for the coating with 8% zinc phosphate, the low-frequency impedance value declined within one day of immersion, but the value remained unchanged after one day. The low-frequency impedance value was fitted in Figure 7a. At the beginning, the low-frequency impedance values of the 0% and 8% zinc phosphate coatings were assessed, and that of the 8% zinc phosphate coating remained after one day of immersion. As immersion time increased, the low-frequency impedance value of the 8% zinc phosphate coating was an order of magnitude higher than the value of the 0% zinc phosphate coating. To study the zinc phosphate coating, the equivalent circuit was fitted in Figure 7b.

**Figure 8:**
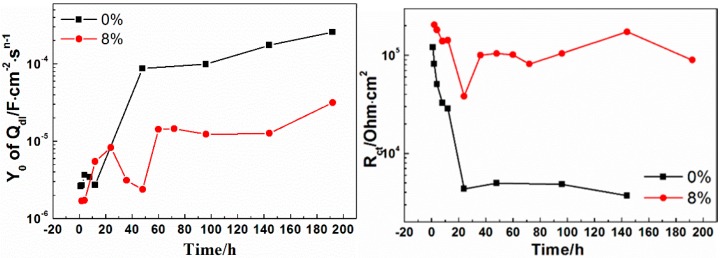
Q_dl_ and R_ct_ values of 0% and 8% zinc phosphate coatings.

Figure 7b shows the equivalent circuit to fit the impedance spectrum. In Figure 7b, R_s_ is the solution resistance, Q_c_ is the coating capacitance, and R_c_ is the coating resistance. Also, Q_dl_ is the capacitance of the double layer between the coating/metal interfaces that can characterize the coating disbonding condition. R_ct_ is the charge transfer resistance. In general, a high R_ct_ reflects low corrosion because the exchange current is directly associated with the electrochemical process of corrosion; therefore, R_ct_ can show the degree of corrosion of the coating. The values of Q_dl_ and R_ct_ are shown in Figure 8. As immersion time increased, the Q_dl_ values of 0% and 8% zinc phosphate coatings increased and the R_ct_ of both decreased, thereby showing serious corrosion between coating/metal interfaces. However, the Q_dl_ value of the 8% zinc phosphate coating was significantly lower than that of the 0% zinc phosphate coating, and its R_ct_ value was significantly higher than the Q_dl_ value of 0% zinc phosphate coating. Therefore, the added zinc phosphate into the coating slowed down the corrosion reaction at the coating defect.

#### 2.3.2. Microscopic Electrochemical Behavior

**Figure 9:**
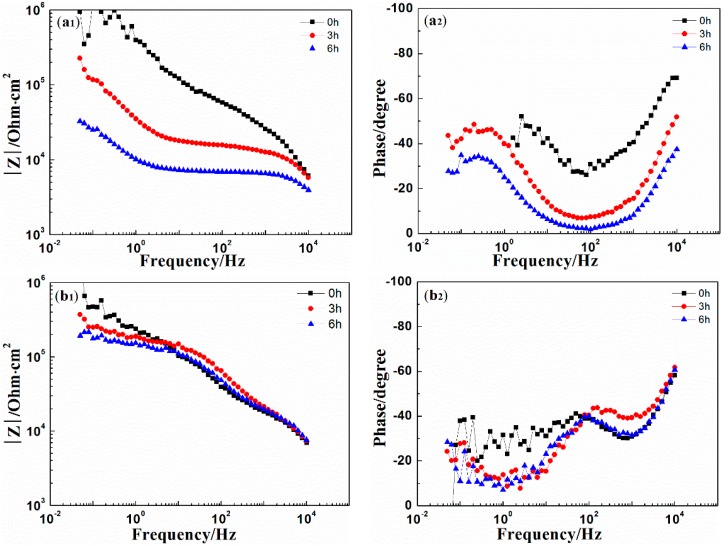
The point measurements of localized EIS (LEIS) of 0% (**a _1_-Nquist**, **a_2_-Bote**) and 8% (**b_1_-Nquist**, **b_2_-Bote**) zinc phosphate coatings immersed in 3.5% NaCl solution.

Figure 9 shows the point measurements of the local alternating current impedance of the (a) 0% and (b) 8% zinc phosphate coating distances from the scratch defect at 1600 µm in 3.5% NaCl solution. After the immersion, the LEIS value of the 0% zinc phosphate coating decreased in 3.5% NaCl, whereas that of the 8% zinc phosphate coating remained relatively stable after immersion for 6 h. The solution penetrated the coating without zinc phosphate and corrosion occurred under the coating. However, the coating with 8% zinc phosphate resisted the medium permeability of the coating.

**Figure 10:**
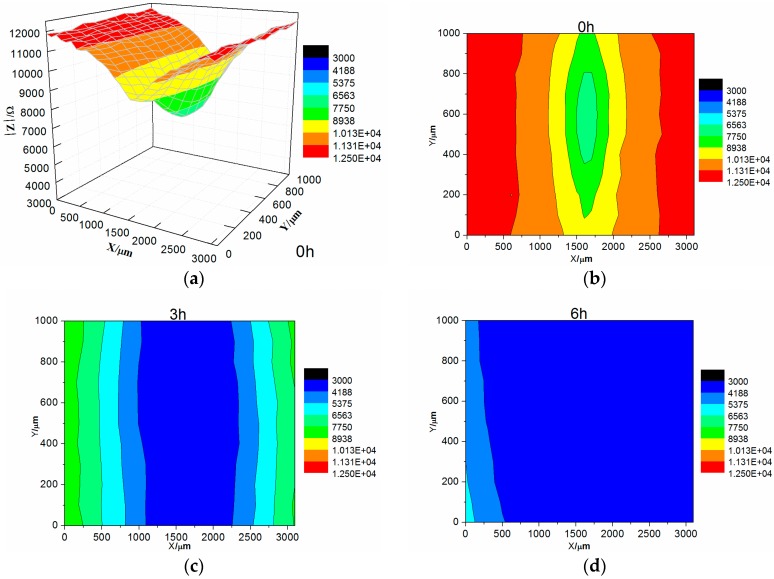
The map measurements of LEIS of the 0% zinc phosphate coating immersed in 3.5% NaCl solution: (**a**) 3D of 0 h; (**b**) 2D of 0 h; (**c**) 2D of 3 h; (**d**) 2D of 6 h.

Figure 10 shows the map measurement of LEIS of the 0% zinc phosphate coating immersed in 3.5% NaCl solution. At the initial stage of immersion, the impedance value of the coating defect in contact with the electrolyte solution was significantly below the impedance value in the intact coating. As immersion time increased, the whole area of impedance was reduced. The impedance modulus value of the coating defect and around dropped rapidly after immersion for 6 h. The coating defect was corroded severely and the area around the coating defect was exfoliated. The solution medium penetrated the bottom of the coating, followed by corrosion.

**Figure 11:**
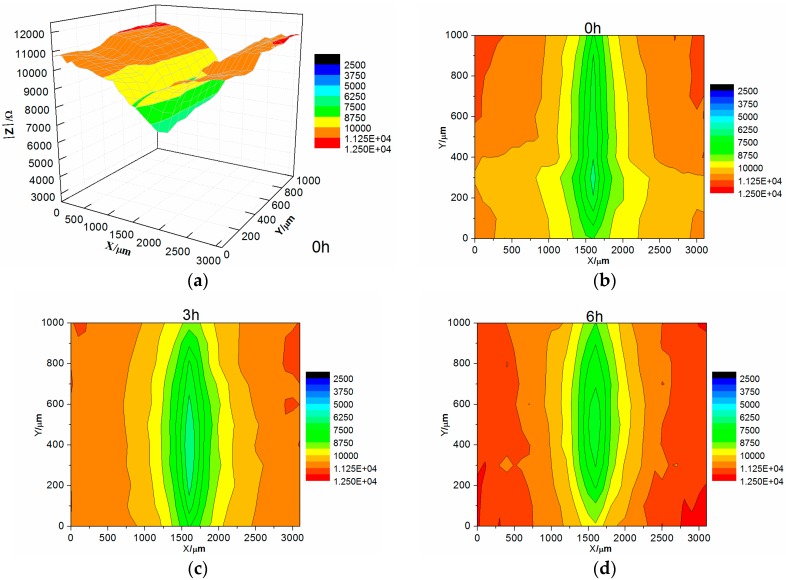
The map measurements of LEIS of the 8% zinc phosphate coating immersed in 3.5% NaCl solution: (**a**) 3D of 0 h; (**b**) 2D of 0 h; (**c**) 2D of 3 h; (**d**) 2D of 6 h.

Figure 11 shows the map measurements of LEIS of the 8% zinc phosphate coating immersed in 3.5% NaCl solution. At the initial stage of immersion, the impedance value of the coating defect was significantly below the impedance value in the intact coating, which was the same as the 0% zinc phosphate coating. As immersion time increased, the impedance of the coating defect did not change and the coating defect around it slightly increased, which was completely different from the 0% zinc phosphate coating. The added zinc phosphate coating prevented the diffusion of corrosive medium in the lateral of coating/metal interfaces and slowed down the corrosion.

**Figure 12:**
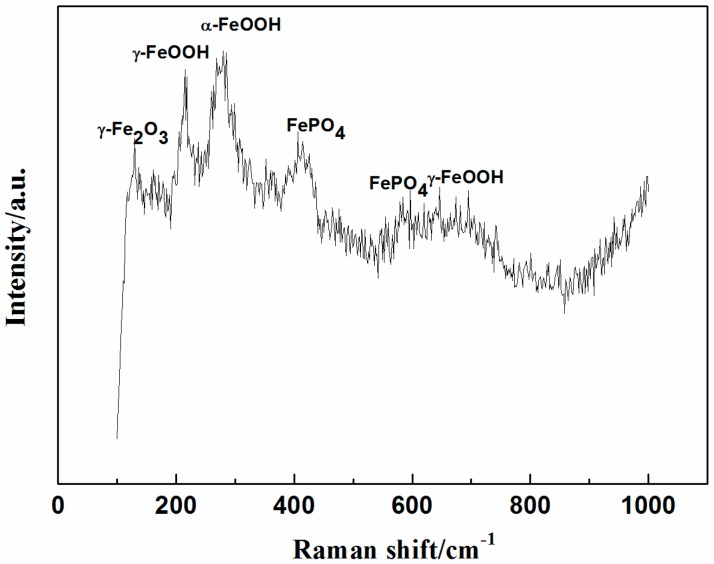
Raman analyses of rust and corrosion product in the defect of the 8% zinc phosphate coating.

After the immersion test in 3.5% NaCl solution, Raman spectroscopy of rust and corrosion product in the defect of the 8% zinc phosphate coating were measured. As shown in Figure 12, the corrosion product was made up by the composition of γ-Fe_2_O_3_, α-FeOOH, γ-FeOOH, and iron phosphate.

## 3. Discussion

In comparing the EIS results of the 0% and 8% zinc phosphate coatings (Figure 6) at an 85 ± 5 µm defects on the coatings, the metals were shown to corrode at the defect areas. At the beginning of immersion, the low-frequency impedance value decreased in both, but as immersion time increased, the low-frequency impedance value of the 8% zinc phosphate coating remained stable at 10^5^ Ohm·cm^2^, which was higher than that of the 0% zinc phosphate (Figure 7a). Moreover, the R_ct_ value of the 8% zinc phosphate coating was significantly higher than that of the 0% zinc phosphate coating (Figure 8a). The added zinc phosphate in the coating slowed down the corrosion reaction at the coating defects. The Q_dl_ value of the 8% zinc phosphate coating was significantly lower than that of the 0% zinc phosphate coating (Figure 8b), showing that the added zinc phosphate slowed down the coating disbonding. The reason for this may be that, when the corrosive medium permeated the coating/metal interfaces, the zinc phosphate was partly dissolved and produced metal substrate phosphate. Zinc phosphate with the composition of γ-Fe_2_O_3_, α-FeOOH, γ-FeOOH, and iron phosphate (Figure 12) can serve as the protective film. The protective film contained carboxyl and hydroxyl groups, which can strengthen the combination of the coating and substrate [29].

(1) $$\mathrm{Fe}\to {\mathrm{Fe}}^{2+}+{2e}^{-}$$

(2) $$O_{2}+{2H}_{2}O+{4e}^{-}\to {4\mathrm{OH}}^{-}$$

(3) $${\mathrm{Zn}}_{3}{\left({\mathrm{PO}}_{4}\right)}_{2}+{2H}_{2}O+{4\mathrm{OH}}^{-}\to 3\mathrm{Zn}{\left(\mathrm{OH}\right)}_{2}+{{2\mathrm{HPO}}_{4}}^{2-}$$

(4) $${\mathrm{Fe}}^{2+}+{{\mathrm{HPO}}_{4}}^{2-}\to {\mathrm{FePO}}_{4}+H^{+}+e^{-}$$

In comparing the LEIS of the 0% and 8% zinc phosphate coatings, the low-frequency impedance value of the 8% zinc phosphate coating defect declined slowly and the 0% zinc phosphate coating defect declined visibly (Figure 9). These results show that the water absorption of the 8% zinc phosphate coating was decreased significantly. When the corrosion medium penetrated the coating/metal interfaces, part of the zinc phosphate dissolved and reacted with Fe to form stable iron phosphates that can block the medium diffusion [30]. 

The impedance values of the 8% zinc phosphate coating increased as the immersion time increased (Figure 11), because the chemical activation of zinc phosphate enhanced the wet coating adhesion. The spread of the corrosive medium in the coating/metal interface was prevented and the coating defect corrosion reaction and disbonding were slowed.

**Figure 13:**
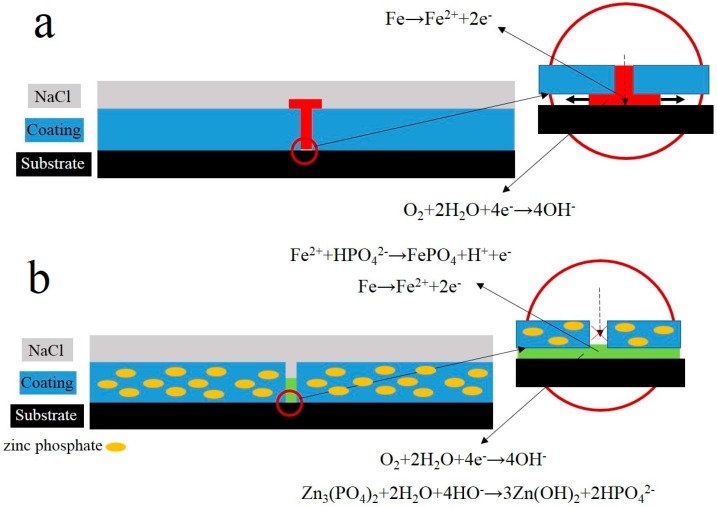
Schematic of the failure mechanism of styrene-acrylic with 0% (**a**) and 8% (**b**) zinc phosphate.

The failure mechanisms of the 0% and 8% zinc phosphate coatings are shown in Figure 13. When the 0% zinc phosphate coating with the defect (Figure 13a) was exposed to corrosive media, the corrosion reaction occurred rapidly on the coating/metal interface and grew suddenly. Furthermore, the coating disbanded from the defect. However, the coating defect with zinc phosphate has two aspects of protection (Figure 13b). First, when the corrosion medium permeates the coating/metal interfaces, the zinc phosphate and Fe generates stable iron phosphates and inhibits the anodic process of corrosion. Second, zinc phosphate can combine with the carboxyl and hydroxyl groups of the coating, through which a stable protective film was formed by the zinc phosphate, coating and metal substrate. All of these reactions can inhibit the cathode reaction from damaging the coating, prevent the corrosion medium from a horizontal diffusion, and slow down the coating defect corrosion and disbonding.

## 4. Materials and Methods

### 4.1. Sample Preparation

**Figure 14:**
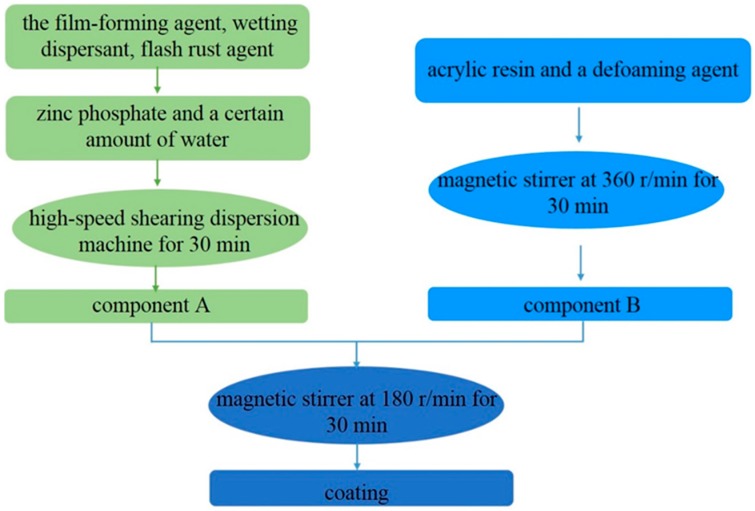
Flowchart to illustrate the process of preparing the coating.

This work used waterborne acrylic resin made in a chemical plant in Beijing, and the zinc phosphate was 800 mesh produced in Guangxi Research Institute of Chemical Industry. A total of 8 wt.% of zinc phosphate composite coating was prepared using high-speed shearing mixing. The zinc phosphate was dried for 15 min at 50 °C, and then the film-forming agent, wetting dispersant, flash rust agent, zinc phosphate, and a certain amount of water were added successively. The above fillers were added and then stirred using a glass bar stirrer. After adding the above fillers, the solution was manually stirred using a glass rod for approximately 10 min and then dispersed using a high-speed shearing dispersion machine (America Red Devil 5400, Trenton, NJ, USA) for 30 min to obtain component A. Acrylic resin and a defoaming agent were mixed manually using a glass rod and then with a magnetic stirrer at 360 r/min for 30 min to obtain component B. Finally, component A was mixed with component B using a glass rod for effective distribution, followed by magnetic stirring at 180 r/min for 30 min to obtain the coating, as shown in the Figure 14.

The metal substrate surface was burnished with a no. 240 sandpaper, cleaned with acetone, and then dried. The above coating was brushed onto the metal substrate surface. The coating thickness for the morphology observation, adhesion test, and EIS was 85 ± 5 µm; whereas, that for the LEIS was 20 ± 2 µm. Both were cured for 15 days at room temperature. The length and width of the coating defects for the EIS test were 10 mm and 120 µm, respectively, and those for the LEIS were 1 mm and 120 µm, respectively, and were implemented by a tool knife made of LB30N steel.

### 4.2. Test Methods

According to ISO4624 standard [31], PosiTestdrawing was used to determine the adhesion strength of the organic coating. The 20-mm diameter dollies were degreased using acetone and then glued to the surface of the coated plates using a two-component epoxy-based cyano-acrylate adhesive. After adhesive curing, a testing apparatus was attached to the loading fixture and was strained at 5 mm/min until the coating material had detached from the substrate.

The micromorphology of the coated section sampled was performed using SEM (QUANTA 250, FEI, Hillsboro, OR, USA). The macromorphology and micromorphology of the coating defects were obtained using a digital camera and stereomicroscope.

Raman spectra from 100 cm^−1^ to 1000 cm^−1^ were used to analyze the composition of the corrosion products of the defect of the 8% zinc phosphate coating. It was measured by the Renishaw DXP780 Raman (Renishaw, London, UK). 

**Figure 15:**
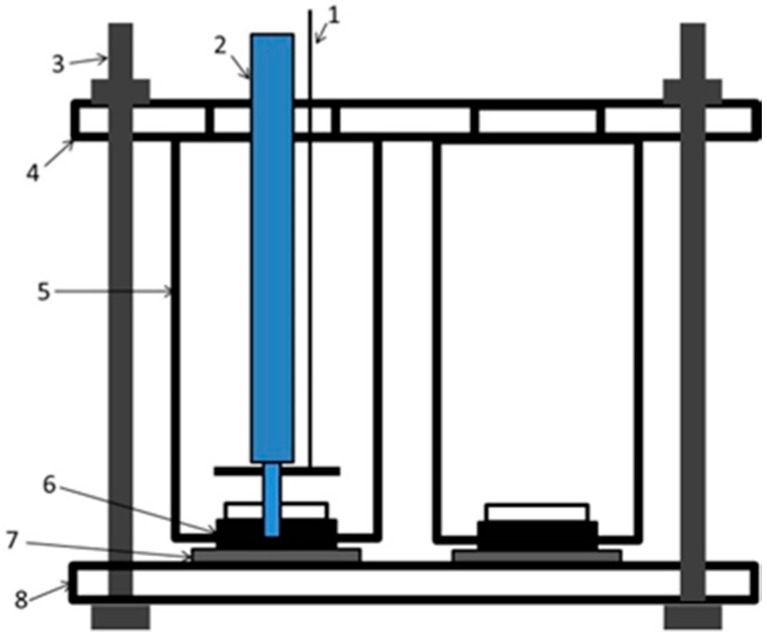
Macroscopic electrochemical test devices.

The macroscopic electrochemical studies on the coating specimens were conducted using a PARSTAT-2273 electrochemical workstation (Princeton Instruments, Trenton, NJ, USA) with an electrolytic cell device as shown in Figure 15. A three-electrode cell system was used in this experiment. The system was assembled with the coating sample as the working electrode, a saturated calomel electrode (SCE) (Rex, Shanghai, China) as the reference electrode, and platinum as the auxiliary electrode. The EIS measurements were taken in a 3.14 cm^2^ area for the coating samples in 3.5% NaCl solution in the frequency range of 100 kHz–0.01 Hz and a disturbance alternating potential voltage of 20 mV. The fitting of EIS data was conducted using ZSimpWin3.2. All EIS tests were conducted at 22 °C.

The LEIS studies on coating specimens were conducted using LEIS components in a M370 micro electrochemical workstation (Bio-Logic, Seyssinet-Pariset, France) The LEIS probe made from a 100-µm diameter platinum wire was used as an auxiliary electrode, the coating sample was the working electrode, and an SCE was used as the reference electrode. The distance between the probe and the sample was approximately 100 µm and the disturbance alternating potential voltage was 10 mV. To ensure the stability of the measurement system, the working electrode was placed in the solution for 20 min until the fluctuation was less than 10 mV. Thereafter, the potential was set as a fixed potential to ensure that the measurement was under open circuit potential. The LEIS used two kinds of test modes, as described below:

Point measurement mode: measurement frequency range was 10,000–0.05 Hz; distance from defects was approximately 1600 µm.

Map scanning mode: testing scope was 3100 × 1000 µm with a step length of 100 µm; according to previous studies [32], 5 kHz was selected to characterize the coating disbonding. 

## 5. Conclusions

Zinc phosphate particles were dispersed evenly in the coating and combined closely with the waterborne acrylic resin. No defects in the coating were found, and the initial adhesion of the coatings with and without zinc phosphate remained. These results showed that zinc phosphate and waterborne acrylic resin have good compatibility. The experimental results of EIS revealed that adding zinc phosphate did not change the electrochemical process of the coating failure but effectively slowed the corrosion degree of the coating defects. The anode and cathode electrochemical processes were characterized via LEIS. Adding zinc phosphate inhibited the anode process of the bare metal in the defect and slowed the corrosion reaction of the anodic metal. Simultaneously, zinc phosphate enhanced the wet adhesion of coating, and was beneficial for preventing the lateral diffusion of the corrosive medium into the coating/metal interface. Moreover, it slowed down the disbonding of the intact coating around the defect. The results showed that adding zinc phosphate to the waterborne acrylic coating can highly improve corrosion resistance.
